# Gestural communication in wild spider monkeys (*Ateles geoffroyi*)

**DOI:** 10.1007/s10071-024-01854-w

**Published:** 2024-03-02

**Authors:** Felipe Villa-Larenas, Miquel Llorente, Katja Liebal, Federica Amici

**Affiliations:** 1 Fundació UdG: Innovació I Formació, Universitat de Girona, Girona, Spain; 2 Departament de Psicologia, Facultat d’Educació I Psicologia, Universitat de Girona, Girona, Spain; 3 Human Biology and Primate Cognition, Institute of Biology, Faculty of Life Science, Leipzig University, Talstraße 33, 04103 Leipzig, Germany; 4 Department of Comparative Cultural Psychology, Max Planck Institute for Evolutionary Anthropology, Leipzig, Germany

**Keywords:** Visual and tactile gestures, Communication, Repertoire size, Intentionality, Platyrrhines

## Abstract

Gestures play a central role in the communication systems of several animal families, including primates. In this study, we provide a first assessment of the gestural systems of a Platyrrhine species, Geoffroy’s spider monkeys (*Ateles geoffroyi*). We observed a wild group of 52 spider monkeys and assessed the distribution of visual and tactile gestures in the group, the size of individual repertoires and the intentionality and effectiveness of individuals’ gestural production. Our results showed that younger spider monkeys were more likely than older ones to use tactile gestures. In contrast, we found no inter-individual differences in the probability of producing visual gestures. Repertoire size did not vary with age, but the probability of accounting for recipients’ attentional state was higher for older monkeys than for younger ones, especially for gestures in the visual modality. Using vocalizations right before the gesture increased the probability of gesturing towards attentive recipients and of receiving a response, although age had no effect on the probability of gestures being responded. Overall, our study provides first evidence of gestural production in a Platyrrhine species, and confirms this taxon as a valid candidate for research on animal communication.

## Introduction

Nonhuman animals (hereafter, animals) communicate with conspecifics in a variety of ways, relying on different forms and mechanisms across multiple modalities, such as tactile, visual, auditory and olfactory ones (Bradbury and Vehrencamp 2011). In several taxa, animals share with humans important characteristics of their vocal communication systems, including aspects of phonology, syntax and vocal learning (Fishbein et al. 2019). Over the last decades, moreover, researchers have provided increasing evidence that also gestures play a central role in the communication systems of several primate species (Call and Tomasello 2007; Cartmill and Maestripieri 2012; Pika and Liebal 2012). Gestures have been defined as discrete physical movements of limbs or head, and body postures, which: 1) are directed to a specific recipient (i.e. a conspecific involved in the communication exchange), 2) are mechanically ineffective (i.e. their action alone could not mechanically produce the response shown by the recipient), and 3) are produced in a goal-directed, intentional way (i.e. implying the accomplishment of specific goals, means-end dissociation, response-waiting, persistence and/or elaboration; see Genty et al. 2009; Hobaiter and Byrne 2011a, 2017; Pika 2008; Tomasello and Call 2007; Tomasello et al. 1985, 1994).

To date, research on primate gestural communication has largely focused on great apes, as manual gestures with communicative purposes were long considered to be rare or absent in other primate species (for a discussion, see Call and Tomasello 2007; Liebal and Call 2012). Important exceptions included studies of gestural communication in small apes (siamangs, *Symphalangus syndactylus*: Liebal et al. 2004) and macaques (e.g. *Macaca spp.*: Gupta and Sinha 2016, 2019; Maestripieri 1996a, b, 1997; Meunier et al. 2013), which evidenced the existence of well-developed gestural communication systems also in species other than great apes. Macaques, for instance, often used gestures and other signals, like facial expressions, to convey information about their emotional states, but they also directed signals to other group members to request participation in different social activities (Maestripieri 1997), with several signals being used by individuals of specific sex and rank categories (Maestripieri 1996a, b). In siamangs, researchers identified around thirty intentionally used signals, including 12 tactile gestures and 8 visual gestures, which individuals flexibly used across different contexts (Liebal et al. 2004).

Moreover, some of these studies evidenced differences across conspecifics in the use of gestures, also depending on the modality in which they were produced. In siamangs, for instance, the number of gesture types produced by males in the tactile modality was on average twice the number of gesture types produced in the visual modality (12:6), whereas females produced a similar number of gesture types in both modalities (8:7; Liebal et al. 2004). Also in red-capped mangabeys (*Cercocebus torquatus*), males had a stronger tendency to produce tactile gesture types, as compared to females (males: 60% in the visual modality, 33% in the tactile modality; females: 71% in the visual modality, 21% in the tactile modality; Schel et al. 2022). These results are in line with studies on great apes, which also showed differences in the use of tactile and visual gestures, with the former being more common in males and younger individuals, than in females and older conspecifics, and visual gestures following an opposite pattern (e.g. Fröhlich et al. 2016; Schneider et al. 2012). Possibly, these differences between sexes or across age reflect differences in the activities in which different classes of individuals engage (e.g. younger individuals are more likely to interact with their mothers, with whom they are often in body contact, and like males they are also more likely to engage in contact play behaviour like sparring and wrestling than older individuals and females — likely implying a higher frequency of tactile gestures; e.g. Beltrán Francés et al. 2020; Soben et al. 2023).

More recently, researchers have started to systematically investigate other important aspects of gestural communication, like repertoire size and intentionality (Freeberg et al. 2012; Prieur et al. 2020). Repertoire size, for instance, measures the number of different gesture types produced by individuals of a given species (Graham et al. 2017; Prieur et al. 2020). For some authors, larger repertoires allow primates more accurate communication and more elaboration when goals are not reached, favouring the establishment and maintenance of more complex social relationships (Roberts et al. 2014; Roberts and Roberts 2019). Repertoire size is known to vary both across and within species. In monkeys, for instance, repertoire size includes 67 visual, tactile and audible gesture types in olive baboons (*Papio anubis*; Molesti et al. 2020), 24 gesture types in Barbary macaques (*Macaca sylvanus*; Hesler and Fischer 2007), and 21 in mangabeys, mostly in the visual modality (Schel et al. 2022). However, direct comparisons of gestural repertoires across species are not necessarily informative, as repertoire size is highly dependent on how gesture types are defined across different species, and on the inclusion of finer-grained distinctions between gesture types. Studies of intra-specific variation, in contrast, do not suffer from these limitations, and suggest differences in repertoire size depending on individuals’ age. Repertoire size, for instance, decreases with age in olive baboons (Molesti et al. 2020), whereas in siamangs it peaks in juveniles to decline in adults (Liebal et al. 2004), as it also happens in great apes (e.g. Hobaiter and Byrne 2011a; Schneider et al. 2012). These longitudinal changes in repertoire size have often been interpreted as individuals first acquiring the fine motor traits necessary to produce gestures during the first years of their development (see Bründl et al. 2021), and then reducing their repertoires through adulthood to the gesture types that are more effective in the specific context they experience (Byrne et al. 2017; Genty et al. 2009; Hobaiter and Byrne 2011a).

Another important aspect of gestural communication systems is intentionality (Freeberg et al. 2012; Prieur et al. 2020). Intentionality can be inferred when individuals produce gestures in the presence of social partners, and account for the recipients’ attentional states as required by the modality in which gestures are produced (Call and Tomasello 2007; Liebal et al. 2007, 2013; Prieur et al. 2020; Roberts et al. 2014). If visual gestures are used intentionally, for instance, they should be more likely when recipients direct their visual attention to the signaller, as they can only be perceived by visually attentive recipients (Call and Tomasello 2007; Liebal et al. 2007). In great apes, several species seem to produce gestures intentionally, despite important variation across individuals (Liebal et al. 2007; Prieur et al. 2020; Tomasello and Call 2019). In chimpanzees (*Pan troglodytes*), for example, older individuals are more likely than younger ones to produce intentional gestures (Fröhlich et al. 2018), and the probability of accounting for recipients’ attentional states increases with age, especially for visual gestures (Amici and Liebal 2022a). For example, while ape infants accounted for recipients’ attentional states in 90% (± 12% SD) of cases of visual gestural production, the ten oldest individuals of the study group did so in all cases (Amici et al. 2022). In contrast, when recipients are visually not attentive, primates may preferentially rely on the production of tactile or auditory gestures, or they can use attention-getting behaviours to attract the recipient’s attention before producing visual gestures (e.g. clapping hands, spitting; see Tomasello and Call 2019).

Studies on intentionality in species other than great apes, however, are scanter. Red-capped mangabeys, for instance, produce the majority of gestures when recipients are visually attentive, and when not, they preferentially rely on auditory or tactile gestures, rather than visual ones (Schel et al. 2022). Similarly, siamangs (Liebal et al. 2004) and olive baboons (Molesti et al. 2020) are more likely to use visual signals when recipients are attentive. In other species, there are no studies on intentionality during spontaneous gestural communication with conspecifics, but experimental studies using food-requesting paradigms suggest that monkeys can also adjust their gestural production to the visual attention of human experimenters. When humans are not visually attending, for instance, monkeys may produce less visual gestures (e.g. tufted capuchin monkeys, *Cebus apella*: Defolie et al. 2015; squirrel monkeys, *Saimiri sciureus*: Anderson et al. 2010), they may increase the frequency of attention-getting gestures (e.g. olive baboons: Bourjade et al. 2014) and gaze alternation (e.g. rhesus macaques: Canteloup et al. 2015), or they may move within the recipient’s visual field (e.g. mangabeys: Aychet et al. 2020; Japanese macaques, *Macaca fuscata*: Castellano-Navarro et al. 2021).

Finally, there might be variation in how effective gestural communication is at achieving the communication goals. In chimpanzees, for instance, the probability of eliciting recipients’ response is higher for older individuals, suggesting that individuals learn through experience how to increase the effectiveness of their communication, by for instance reducing the frequency of gestural sequences and/or better accounting for others’ attentional states (Amici and Liebal 2022a; Hobaiter and Byrne 2011b). Moreover, visual gestures may be more effective if they are preceded by attention-getters, which are audible signals that beyond serving a communicative function per se might also be used to attract the attention of inattentive recipients towards the gesturing individual (see Tomasello and Call 2019). In monkeys, there are no systematic studies yet on the factors affecting the effectiveness of gestural communication. However, the percentage of gestures that elicit a response by recipients appears to be similar to that of apes, with 63% of gestures being responded in chimpanzees, 62% in orangutans (*Pongo abelii*), 66% in siamangs (see Amici and Liebal 2022a), and 65% in red-capped mangabeys (Schel et al. 2022).

Here, we provide a first assessment of the gestural communication systems of a Platyrrhine species, to contribute to the study of the evolutionary origins of communication systems. For this purpose, we observed a wild group of 52 Geoffroy’s spider monkeys (*Ateles geoffroyi*) and assessed individual variation in the probability of producing visual and tactile gestures, in the size of individual repertoires, and in the probability of accounting for receivers’ attentional state (as a form of intentionality) and receiving a response (as a form of effectiveness) when producing gestures. Spider monkeys are an ideal model to study gestural communication, as they live in complex socialities similar to those of chimpanzees, which are characterized by high levels of fission–fusion dynamics (i.e. individuals frequently split and merge again into subgroups of varying size and composition; Aureli et al. 2008). For some authors, high levels of fission–fusion might favour the emergence of larger repertoires, which would allow individuals to more effectively deal with the dynamic sociality in which they live (Aureli et al. 2008).

First, we hypothesized that the probability of using tactile and visual gestures would vary across individuals depending on their sex and age (Table 1). In particular, based on literature in other species (e.g. Fröhlich et al. 2016; Liebal et al. 2004; Schel et al 2022; Schneider et al 2012), we predicted that tactile gestures would be more likely produced by males (Prediction 1a) and younger individuals (Prediction 1b), as compared to females and older individuals; in contrast, we predicted that visual gestures would be more likely produced by females (Prediction 2a) and older individuals (Prediction 2b), as compared to males and younger individuals. Moreover, it is possible that the use of tactile gestures or visual gestures depends on the functional context: while spider monkeys might rely more on the visual modality during travelling, when individuals are likely spread and contact may be difficult, tactile gestures might be more likely during social interactions, when physical contact is not only possible but also likely to be expected. As there are no studies yet assessing how gesture types are used in different contexts by this species, however, we refrained from making specific predictions.

**Table 1:** Predictions of the study, model used to test them, and whether they were confirmed

Predictions		Model	test predictors	Confirmed?
1	Males (1a) and younger monkeys (1b) produce more tactile gestures than females and older monkeys	1	~ sex, age, subgroup activity	1a: no 1b: yes
2	Females (2a) and older monkeys (2b) produce more visual gestures than females and older monkeys	2	~ sex, age, subgroup activity	2a: no 2b: no
3	Repertoire size varies through age, first increasing and then decreasing (3)	3	~ age (squared)	3: no
4	Probability of producing gestures towards attentive recipients increases with age, especially in the visual modality (4a), and if gestures are preceded (but not followed) by a vocalization, especially in the visual modality (4b)	4	~ modality*age, modality*vocal. before, modality* vocal. after	4a: yes 4b: yes (but across modalities)
5	Probability of gestures being responded increases with age (5a), and if gestures are preceded (but not followed) by a vocalization, especially in the visual modality (5b)	5	~ age, modality*vocal. before, modality*vocal. after	5a: no 5b: yes (but across modalities)

We further hypothesized inter-individual variation in repertoire size (Table 1). We predicted that repertoire size would first increase during the very first years of monkeys’ development, and then decrease during adulthood (Prediction 3), as in other species (Hobaiter and Byrne 2011a; Liebal et al. 2004; Schneider et al. 2012).

Furthermore, we hypothesized that the probability of producing gestures towards attentive recipients would vary depending on gesture modality, signallers’ age and the use of attention-getters (Table 1). In particular, we predicted that older individuals would be more likely than younger ones to account for recipients’ attentional state, but only/more when gestures were produced in the visual modality (Prediction 4a). Moreover, we predicted that the probability of producing gestures towards attentive recipients would increase if gestures were preceded (but not followed) by a vocalization, but only/more when gestures were produced in the visual modality (Prediction 4b).

Finally, we hypothesized that the probability of receiving a response would vary depending on signallers’ age and the use of attention-getters (Table 1). In particular, we predicted that gestures would be more likely responded when produced by older individuals, as compared to younger ones (Prediction 5a), and if preceded (but not followed) by a vocalization, but only/more when gestures were produced in the visual modality(Prediction 5b).

## Methods

*Ethics.* Permission to conduct the study was granted by the Mexican institutions CONANP (Comision Nacional de Areas Naturales Protegidas) and SEMARNAT (Secretaría de Medio Ambiente y Recursos Naturales). Our study complied with the Principles for the Ethical Treatment of Nonhuman Primates by the American Society of Primatologists (2001).

*Field site and study subjects.* We conducted the study in the natural protected area Otoch Ma’ax Yetel Kooh in Yucatan, Mexico (20° 38ʹ N, 87° 38ʹ W), which includes old-growth, semi-evergreen medium forest and 30–50-year-old successional forest (Ramos-Fernández and Ayala-Orozco 2003). We observed a group of 52 well-habituated Geoffroy’s spider monkeys, including 14 adult females, 9 adult males, 3 subadult females, 2 subadult males, 9 juvenile females, 6 juvenile males, 4 infant females and 5 infant males (i.e. infants: < 2 years; juveniles: 2–5 years; subadults: 6–7 years; adults: > 8 years; see Shimooka et al. 2008; Soben et al. 2023; Table 2). In contrast to juveniles, infants are highly dependent on their mothers, which frequently nurse, carry and are in body contact with them; however, both infants and juveniles typically travel with their mothers and join the same subgroup (Shimooka et al. 2008; Soben et al. 2023). Given the relatively large group size of our study sample (which included several individuals of different age class and sex) and its high levels of fission–fusion dynamics (which allow group members to merge with different partners into the same subgroup), individuals in our study group had the opportunity to interact with many different partners of different sex and age. All monkeys could be individually recognized through facial features and differences in fur coloration, and their age was determined through demographical records collected over several years.

**Table 2:** List of study subjects for each sex and age class. In parentheses, we report the number of different gesture types produced by each subject, out of the total number of gestures produced during the study

Sex	Age	Study subjects
Female	Infant	Chikich (17/52), Corona (0/0), Selva (6/9), Yuli (14/65)
	Juvenile	Aura (10/24), Braga (14/50), Canela (8/22), Eek (13/32), Ixchel (9/19), Luna (15/39), Luz (7/11), Sacbe (11/31), Yalit (20/80)
	Subadult	Bekech (1/2), Morita (10/29), Nit (7/10)
	Adult	Antena (1/2), China (7/14), Flor (0/0), Ikil (7/8), Joanne (5/12), Lola (7/8), Mandíbula (4/8), Marylin (4/6), Mich (8/11), Pancha (21/38), Rwanda (5/8), Tanga (14/28), Verónica (3/3), Xibalba (7/9)
Male	Infant	Alma (1/1), Cacao (6/16), Chaac (11/44), Covid (13/37), Sol (4/5)
	Juvenile	Fabrizio (15/32), Jesus (0/0), Pekín (24/108), Poncho (15/45), Puma (13/44), Voldemort (15/51)
	Subadult	Nacho (15/29), Valentín (13/37)
	Adult	Andrés (11/31), Apolo (8/10), Boxhuevos (14/28), Digit (13/20), Eulogio (11/26), Juan (13/28), Marcos (8/15), Sancho (11/34), Wiguiberto (12/25)

**Fig. 1:**
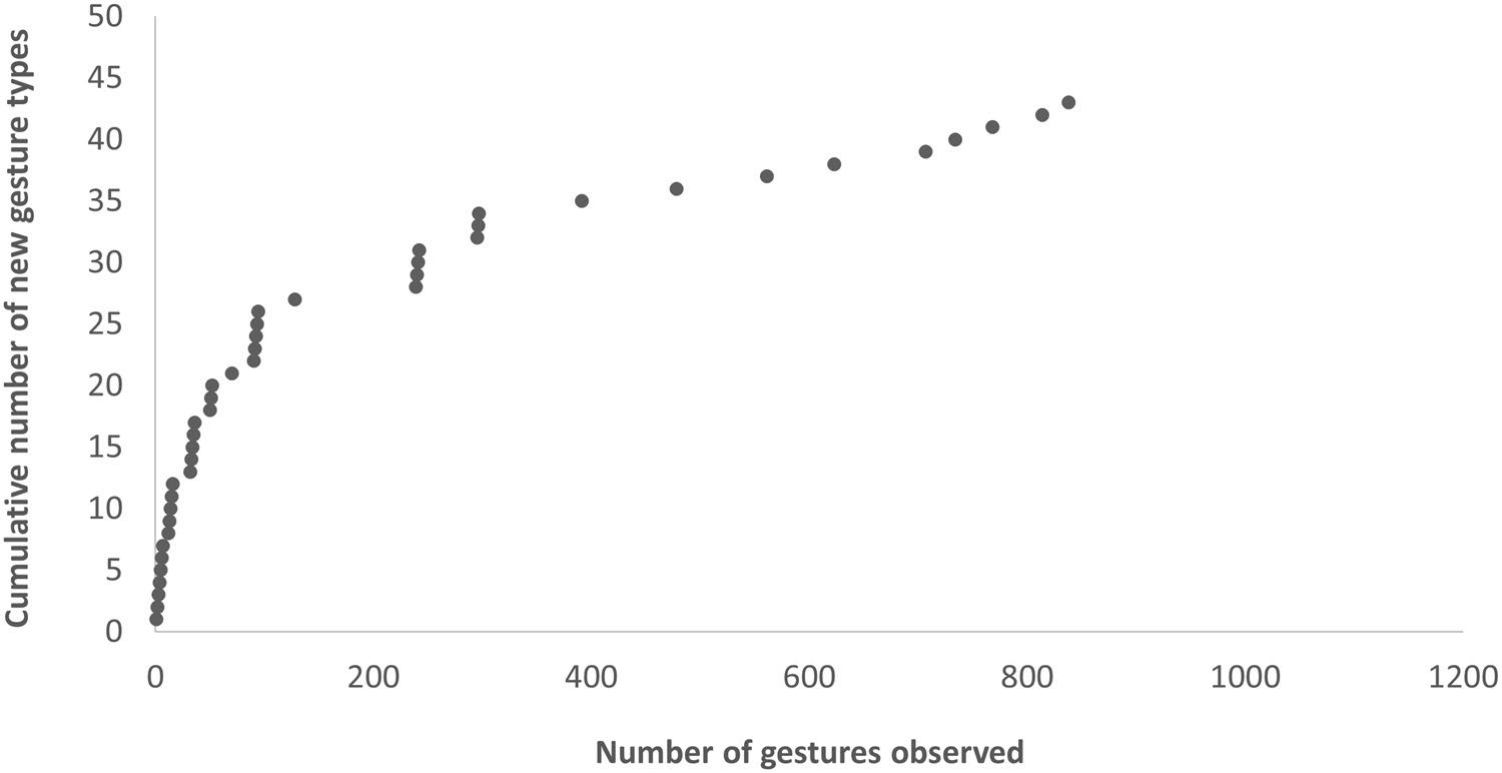
Cumulative number of new gesture types being observed in the study group, as a function of the number of gestures observed

*Data collection.* We collected data from January to June 2022, for 5 days a week, from 06:00 to 13:30. In the first 2 months, monkeys were observed ad libitum by the first author, to prepare an inventory of all the gestures exhibited by the individuals in the study group. The first author categorized as potential gestures all the discrete physical movements of limbs or head and all the body postures observed, which: 1) were directed to a specific recipient, 2) were mechanically ineffective actions, and 3) were produced in a goal-directed, intentional way (; see Genty et al. 2009; Hobaiter and Byrne 2011a, 2017; Pika 2008; Tomasello and Call 2007; Tomasello et al. 1985, 1994). The first author first described these potential gestures, by specifying as applicable the position of arms, hands, fingers and/or body also relatively to the recipient, and the context in which they usually were produced. We then compared these potential gestures to the gestural categorizations currently used in other primate species (e.g. Hobaiter and Byrne 2011a; Liebal et al. 2004, 2006) and to the definitions and ethograms used in literature on spider monkeys (e.g. Schaffner et al. 2012). These comparisons to literature allowed us to further refine categories when needed (e.g. to differentiate between gesture types like arm wrapping and embrace, or tap and touch, which can have a different function despite having a similar form), or to merge them (e.g. if formal differences described by the first author appeared to be functionally irrelevant, and likely reflected mere formal variation of the same gesture type, rather than different gesture types). Finally, if these potential gestures were seen at least twice in the study group, they were included in our ethogram, which ended up including 43 different gesture types (see Table 3 for the complete list of gestures and definitions). For the cumulative number of new gesture types observed in the study group as a function of the observational effort, please refer to Fig. 1.

**Table 3:** List of gesture types observed with their definition, the modality (V = visual, T = tactile, A = auditory) and context (1 = affiliative, 2 = agonistic, 3 = feeding/foraging, 4 = fusion, 5 = resting, 6 = sex, 7 = social play, 8 = solitary play, 9 = travelling) in which they usually happened, the response they usually elicited (i.e. accept sniff, approach, arm wrapping, body contact, climb, dangle, embrace, embrace tail, groom, move away, nurse, play, sex, stop previous activities, submission), preceded by the number of gestures followed by a clearly visible response, the number of times they were observed (N) and number of individuals producing it (I) during this study; contexts are ordered so that the most frequent ones are listed first, and the second most frequent ones are listed in parentheses; responses are ordered so that the more frequent ones come first; although most gestures types could only be produced in one modality, seven gesture types were usually produced in one modality but in few specific cases were produced in other modalities, so that the second modality is also included in parentheses in the respective column (e.g. Big loud scratch, Gallop, Leaf clipping and Stomp were usually produced in the visual modality, but in few cases they were also audible and in those cases they were thus considered to be produced in the auditory modality)

Gesture	Definition	Modality	Context	Response	N	I
Arm shake	The actor shakes his arm, repeatedly moving it back and forth	V	1	2: Move away	4	2
Arm wrapping	The actor wraps one arm around the recipient’s back, who also wraps the other’s back, while maintaining physical contact and aggressively facing forward towards a third party	T	2	4: Arm wrapping	6	3
Beckon	The actor moves his hand in an upward sweep, from the elbow or wrist towards himself	V	9 (1,3,6,7)	5: Climb, Play	6	5
Big loud scratch	The actor exaggeratedly scratches his own body with strong scratching movements	V (A)	1,2,5,7	2: Move away	4	3
Bipedal stance	The actor has a bipedal posture, often holding arms out laterally and turning the back to the recipient	V	7 (2,5)	5: Play	7	6
Bite	The actor gently bites the recipient’s body with his lips or teeth	T	7 (2,8)	110: Play, Move away	126	26
Body shake	The actor repeatedly shakes his whole body in the direction of the recipient	V	1,7	0	2	2
Bow	The actor bends forward from the waist, while standing upright	V	7	1: Stop	2	2
Dangle	The actor hangs from a branch above another individual, using one or both arms or the tail	V	7 (1)	93: Play, Dangle, Move away	128	31
Dangle shake	The actor hangs from a branch above another individual, using one or both arms or the tail, while (repeatedly) shaking his body	V	7	1: Play	4	2
Embrace	The actor wraps one or both arms around the recipient’s back or neck, while maintaining physical contact and facing each other	T	1 (4)	64: Embrace, Accept sniff	73	28
Embrace tail	The actor wraps his tail around the recipient’s tail while facing each other, so that the two tails are intertwined with each other	T	5 (1)	12: Embrace tail	13	12
Frontal threat	The actor leans in the direction of the recipient, extending his back while being supported by one lower limb and one upper limb (sometimes also the tail), whereas the other limbs are free	V	2 (3,7)	16: Submission, Move away	26	13
Gallop	The actor makes exaggerated running movements, so that the contact of his hands and feet on the branches is clearly audible	V (A)	7 (2)	112: Play, Move away	117	28
Grab/ Grab hold	The actor holds his hand firmly closed over the recipient’s body (if longer than 2 s, it is considered a Grab hold)	T	7 (6)	147: Play, Move away	169	33
Grab pull	The actor holds his hand firmly closed over the recipient’s body, but force is exerted to move the recipient from his position	T	7 (1)	49: Play, Move away	57	30
Hand on	The actor places his hand on the recipient (typically his palm), and maintains contact for at least 2 s	T	1 (2,3)	5: Body contact, Move away	6	6
Hand shake	The actor repeatedly moves the hand back and forth, from the wrist	V (T)	7 (1,2)	3: Play	5	4
Head butt	The actor briefly and firmly pushes his head towards the recipient’s body	T	1 (5)	9: Nurse	12	6
Head shake	The actor shakes his head, repeatedly moving it back and forth	V	7 (8)	5: Play, Approach	7	6
Hit other	The actor moves his whole arm, leading to a brief but hard contact of the closed fist with the recipient’s body	T	7 (2)	33: Play, Move away	36	19
Leaf clipping	The actor tears strips of a leaf/leaves off with the teeth or mouth, which he holds in his hand, thereby producing a flashy sound	V (A)	7	0	3	2
Object shake	The actor shakes an object, repeatedly moving it back and forth, while looking at the recipient	V	2,3	2: Move away	3	2
Open mouth	The actor opens his mouth, by lowering the lower lip and raising the upper lip with short repetitive movements, and teeth may become visible	V	2 (6,7)	3: Move away, Stop	6	5
Pectoral sniff	The actor places his head in the area of the recipient’s chest-axilla	T	4 (1)	45: Accept sniff	50	22
Pirouette	The actor turns around the vertical axis of his body	V	7	1: Play	3	2
Poke	The actor firmly and briefly pushes one or more fingers on the recipient’s body	T	7 (5)	13: Play	22	14
Pounce	The actor moves through the air to land quadrupedally on the recipient’s body	T (V)	7 (2)	61: Play, Move away	72	25
Present climb	The actor (usually a mother) extends his arm or leg to an immature to facilitate climbing onto his body	V	3 (9)	9: Climb, Play	9	5
Present genitals	The actor (usually a female) approaches the recipient (usually a male) from behind, exposing the swelling or anus to the recipient’s attention	V	6	3: Sex	4	2
Present grooming	The actor exposes an area of his body to the recipient’s attention, as if soliciting grooming	V (T)	1 (5)	79: Groom	99	35
Pull tongue	The actor sticks out his tongue in the direction of the recipient	V	5,7	0	3	2
Push	The actor puts the palm of his hand in contact with the recipient’s body, exerting force in an attempt to displace the recipient	T	7 (1)	11: Play, Move away	16	12
Roll over	The actor rolls onto his back exposing his stomach, usually while repeatedly making arm and/or leg movements	V	7	1: Play	2	2
Shake hands	The actor grasps the recipient’s hand with his own hand, and then makes small repeated movements back and forth from the wrist	T	7 (5)	11: Play	13	13
Slap	The actor moves his arm from the shoulder, so that the hand or fingers come in short but hard contact with the recipient	T	7 (2)	35: Play, Move away	39	19
Somersault	The actor curls his body into a compact position on the floor and rolls forward, so that his feet go over his head before he returns to a seated position	V	7	2: Play, Move away	3	2
Stiff walk	The actor walks quadrupedally with slow exaggerated movements	V	7	1: Play	3	2
Stomp	The actor vertically lifts one foot/both feet and brings the sole into brief audible contact with the surface on which it rests (i.e. ground or branch)	V (A)	7 (5)	6: Play, Move away	11	8
Tandem walk	The actor puts his arm over the recipient’s body and both walk forward while maintaining this position, often in a play context to recruit other players	T	2,7	1: Play	2	2
Tap other	The actor moves his arm from the wrist or elbow, leading to a repetitive firm short contact between his fingers and the recipient’s body	T	7 (9)	6: Play, Move away	7	3
Touch	The actor places his hand or fingers on the recipient, maintaining contact for less than 2 s	T	7 (1)	3: Play, Move away	6	5

From March to June 2022, we conducted 15‐min focal animal samples with continuous sampling (Altmann 1974), for a total of 551 focal samples (mean ± SD: 2.8 ± 0.6 h per subject). We observed all the individuals in the group on a pseudorandomized basis (i.e. starting focal observations from the first individual on a list where all the individuals were randomly ordered). We recorded focal samples with CyberTracker on mobile devices (Blackview BV9700 PRO, Runbo F1 4G 5.5), with one to two observers dictating and the third one writing into the device. We started data collection only after the observers reached 80% inter-observer reliability for the coded behaviours (see below). During the focal samples, we recorded all the gestures produced by the focal individual, the subgroup main activity during the focal observation (i.e. feeding/foraging, resting, travelling, social interactions and other behaviours, the latter including subgroup activities that were rarely recorded, like fission–fusion events), which was one during each focal sample, and the exact duration of the focal observation (i.e. removing the time in which the individual was out of view), beyond other information on social interactions that were used for other studies.

Whenever a gesture occurred, we recorded: (i) the gesture type produced; (ii) the identity of the monkey producing the gesture (i.e. signaller), and (iii) the identity of the monkey to which it was directed (i.e. recipient); (iv) the functional context in which the gesture was produced (which, by only referring to the signaller and recipient, could be more detailed than the subgroup activity, and included: feeding/foraging, resting, travelling, affiliative interactions, sexual interactions, agonistic interactions, social play, solitary play, fusion events); (v) the recipient’s response (i.e. whether they reacted by changing their behaviour, and/or looking at the focal individual, within 5 s from the gesture); (v) the recipient’s attentional state (i.e. whether they had direct eye with the focal individual, or their body was oriented towards the focal and this was in their field of vision, and their attention was not distracted by other individuals or events); (vii) whether the gesture was tactile or visual (i.e. whether the gesture implied physical contact or not); and (viii) whether the focal individual also produced a vocalization within the 2 s preceding the gesture, and (ix) within the 2 s following the gesture. Coding both functional context and recipient’s response was not redundant, as it allowed us to differentiate between the activity in which the signaller engaged right before gesturing, and the reaction that the gesture triggered in the recipient. For instance, if the signaller was playing alone when gesturing, and the recipient responded to the gesture by starting a social play session, we coded context as solitary play, and response as social play. When the recipient responded to the gesture and the response was clearly visible, we described the recipient’s response and assigned it to one of the following categories, which were based on studies in other primate species (e.g. Hobaiter and Byrne 2014) and adjusted during the first 2 months of the study based on our observations of the study group: accept sniff, approach, arm wrapping, body contact, climb, dangle, embrace, embrace tail, groom, move away, nurse, social play, sex, stop previous activities, submission. Moreover, we collected ad libitum all visible instances of gestures occurring in the group, also coding the behaviours above (i-ix). This resulted in 880 gestures recorded during focal observations, and further 306 gestures recorded during ad libitum data collection, for which we could also record the behaviours above (i-ix).

*Statistical analyses.* We ran generalized linear mixed models (Baayen et al. 2008) in R (R Core Team 2020), using the package glmmTMB (Brooks et al. 2017). We ran the first two models to assess inter-individual variation in the probability of producing gestures in the tactile and visual modalities. For this purpose, we only used the gestures recorded during focal observations, for which observational effort could be controlled for — something that was necessary to account for the fact that study subjects were observed for different amounts of time. In the dataset, we entered one line for each focal observation (*N* = 551). Our binomial response was whether the focal subject produced at least one gesture in the tactile modality (Model 1) or in the visual modality (Model 2) during the focal observation. Therefore, if an individual produced at least one tactile gesture and at least one visual gesture in the same focal observation, this was entered as a positive response in both Model 1 and Model 2. In both models, we included as test predictors the focal individual’s sex and age (as a continuous variable, in years), and the subgroup main activity during the focal observation. This allowed us to test our predictions about possible sex- and age-differences in the probability of using tactile and visual gestures (Predictions 1–2 in Table 1). As offset term we further included the duration of the focal observation, and as random factor the focal individual’s identity.

Models 3 to 5, in contrast, provided information on variation in repertoire size, probability of accounting for receivers’ attentional state (as a form of intentionality) and probability of receiving a response (as a form of effectiveness). In these models, we did not include sex as test predictor (but only as control), because we did not expect sex differences in the responses. Model 3 assessed individual variation in repertoire size. In the dataset, we entered one line for each study subject that was observed gesturing more than once during the study (*N* = 48). We operationalized repertoire size as the number of different gesture types produced by each study subject throughout the study (including gestures observed during focal observations and ad libitum), which we modelled with a Poisson distribution. We included as test predictors the individual’s age (also as squared term, as the relation between repertoire size and age might not be linear), and as control the individual’s sex and the cumulative number of gestures observed for that individual. Including the last control allowed us to measure the variety of different gesture types used, while accounting for the fact that individuals were not observed for the same amount of time and differed in the frequency with which they gestured. Including the duration of focal observations as offset term was instead not possible, as our dataset included gestures observed during focal observations and also ad libitum. Removing this control from the models led to similar results (i.e. no difference between full and null model; see below). Model 3 therefore allowed us to test our prediction that repertoire size would vary through age, first increasing and then decreasing (Prediction 3 in Table 1), while accounting for the cumulative number of gestures observed.

The dataset for Models 4 and 5 included one line for each gesture observed (*N* = 1186). As we analysed the specific characteristics of gestures (i.e. whether they were produced when others were attentive, and were responded to), and not their distribution, we did not have to include observational effort in these models and we could include both gestures collected ad libitum and with focal observations. Model 4 assessed whether the recipient was attentive when a gesture was produced, and whether this was influenced by the signaller’s age, gesture modality and the use of vocalizations (before/after the gesture). Our binomial response was whether the recipient was attentive when the gesture was produced. As test predictors, we included the three 2-way interactions of gesture modality (i.e. tactile or visual) with signaller’s age, with a binomial variable measuring whether the gesture was preceded by a vocalization, and with a binomial variable measuring whether the gesture was followed by a vocalization. These interactions allowed us to test our prediction that older individuals would be more likely than younger ones to account for recipients’ attentional state when producing visual gestures, and that recipients would be more likely attentive to gestures in the visual (but not in the tactile) modality that were preceded (but not followed) by a vocalization (Prediction 4 in Table 1). In this model, we also controlled for signaller’s sex, recipient’s sex and age, and for the context in which the gesture was produced, entering the signaller’s and recipient’s identities as random factors. Including these controls allowed us to account for the fact that our datapoints were not equally distributed depending on these variables, as for instance we did not have the same number of observations for each context or recipient’s sex and age. This, however, might be problematic, because in some contexts, for instance, it might be logistically more challenging for individuals to account for others’ visual attention (e.g. when travelling).

In Model 5, we finally assessed whether the probability of receiving a response depended on signaller’s age and on the use of vocalizations before the gesture, especially in the visual modality. Our binomial response was whether the recipient responded to the gesture, and the test predictors, controls and random factors were identical to Model 4 (except that we included signaller’s age as main term, instead of its interaction with modality, as we did not expect gesture modality to mediate the link between signaller’s age and probability of receiving a response). In particular, as test predictors we included signaller’s age and the two 2-way interactions of gesture modality (i.e. tactile or visual) with a binomial variable measuring whether the gesture was preceded by a vocalization, and with a binomial variable measuring whether the gesture was followed by a vocalization. This allowed us to test our prediction that gestures would be more likely responded when produced by older signallers and when preceded (but not followed) by a vocalization, especially in the visual modality (Prediction 5 in Table 1). In this model, we also controlled for signaller’s sex, recipient’s sex and age, and for the context in which the gesture was produced, entering the signaller’s and recipient’s identities as random factors.

We *z*-transformed all continuous predictors (i.e. age, number of gestures observed) to facilitate model convergence and interpretation of model coefficients. We used likelihood ratio tests to compare each of the full models described above to a null model, which was identical to the full one but did not include the test predictors (Dobson and Barnett 2018). In case of a significant difference between the full and the null model, we used the drop1 function to assess which test predictors were significant. In case interactions were not significant, the model was re-run after removing the non-significant interactions and entering their terms as main effects. In case of significant categorical predictors with more than two categories (i.e. context), we used the emmeans package to run post-hoc comparisons with Tukey adjustments (Lenth 2020). Below we only report significant post-hoc comparisons; all other comparisons had a *p* value > 0.05. We checked model assumptions with the “DHARMa” package (Hartig 2022), including residual diagnostics and overdispersion. We used the “performance” package (Lüdecke et al. 2021) to check for multicollinearity, which was low (maximum variance inflation factors across models = 2.87; Miles 2005).

## Results

*Individual variation in the probability of using tactile and visual gestures.* The percentage of gestures produced in the tactile modality was 56% in females (i.e. 338 occurrences) and 58% in males (i.e. 335 occurrences). Through age, the percentage of gestures produced in the tactile modality decreased, reaching 62% in infants (i.e. 42 occurrences), 57% in juveniles (i.e. 399 occurrences), 60% in subadults (i.e. 65 occurrences) and 55% in adults (i.e. 167 occurrences). Whereas 27% of the focal observations included at least one tactile gesture (i.e. 149/551 focal observations), 30% of the focal observations included at least one visual gestures (i.e. 163/551 focal observations). Focal observations in which at least one tactile gesture was produced were conducted when the subgroup main activity was feeding/foraging (i.e. 64 occurrences), resting (i.e. 40 occurrences), travelling (i.e. 13 occurrences), social interactions (i.e. 24 occurrences) and other behaviours (i.e. 8 occurrences). Focal observations in which at least one visual gesture was produced were conducted when the subgroup main activity was feeding/foraging (i.e. 56 occurrences), resting (i.e. 60 occurrences), travelling (i.e. 8 occurrences), social interactions (i.e. 27 occurrences) and other behaviours (i.e. 12 occurrences).

**Fig. 2:**
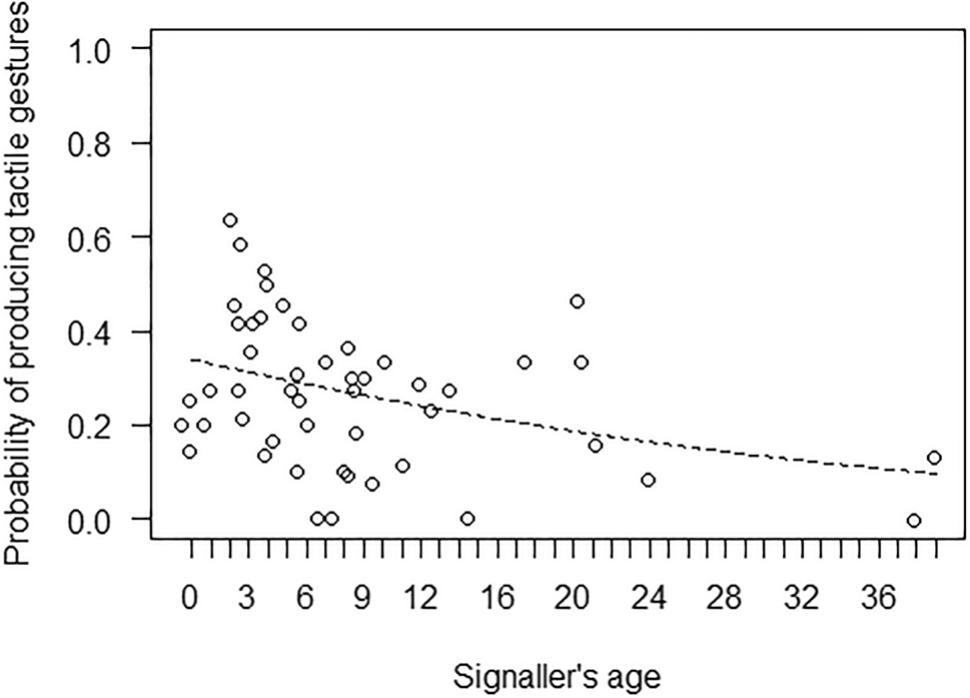
Probability of producing at least one gesture in the tactile modality during the 15-min focal, as a function of the signaller’s age (in years; infants: < 2 years; juveniles: 2–5 years; subadults: 6–7 years; adults: > 8 years). Circles represent the mean probability of producing tactile gestures for each signaller (*N* = 49), after aggregating the data points used for Model 1. The line represents the fitted model, which was like Model 1 but unconditional on all the factors that were standardized, and with observational effort expressed in 15-min intervals. The probability of producing tactile gestures was significantly higher for younger than older individuals

**Fig. 3:**
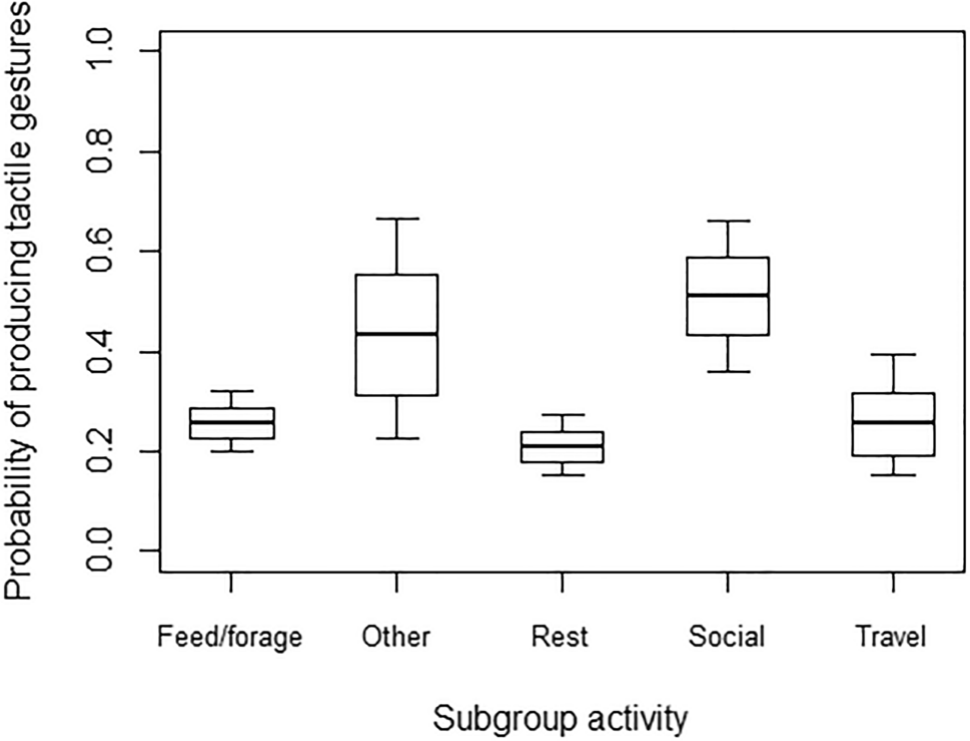
Probability of producing at least one gesture in the tactile modality during the 15-min focal, as a function of subgroup activity. The thick lines of the box plots represent the mean probabilities for each subgroup activity, as estimated by the fitted model, which was like Model 1, but unconditional on all the other factors that were standardized. The ends of the boxes represent the estimated standard errors, and the ends of the whiskers represent the 95% confidence intervals. The probability of producing tactile gestures was significantly higher when the subgroup was mainly engaged in social interactions, rather than feeding/foraging or resting

**Fig. 4:**
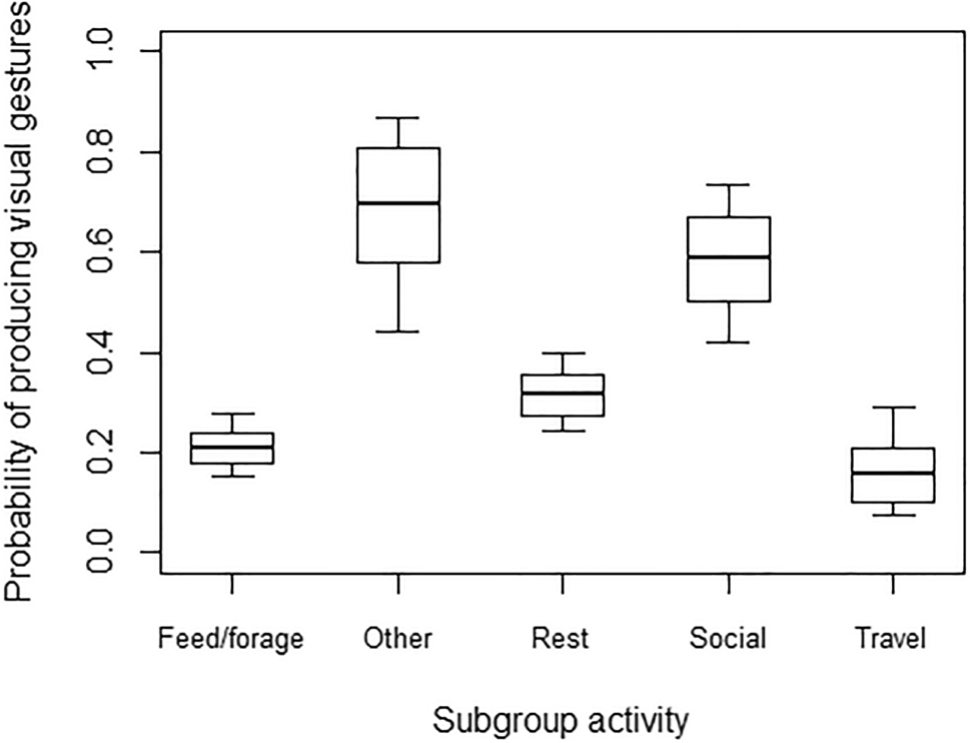
Probability of producing at least one gesture in the visual modality during the 15-min focal, as a function of subgroup activity. The thick lines of the box plots represent the mean probabilities for each subgroup activity, as estimated by the fitted model, which was like Model 2, but unconditional on all the other factors that were standardized. The ends of the boxes represent the estimated standard errors, and the ends of the whiskers represent the 95% confidence intervals. The probability of producing visual gestures was significantly higher when the subgroup was mainly engaged in social interactions or other behaviours, as compared to feeding/foraging, resting or travelling

In Model 1, the full model significantly differed from the null model, with both age and subgroup activity having a significant effect (Table 4). In particular, the probability of using at least one tactile gesture during the 15-min focal was higher for younger than older individuals (Fig. 2). Moreover, post-hoc tests showed that the probability of using at least one tactile gesture during the focal was also higher when the subgroup was mainly engaged in social interactions, rather than feeding/foraging (*p* = 0.013) or resting (*p* = 0.001; Fig. 3). In Model 2, the full model significantly differed from the null model, but only subgroup activity had a significant effect (Table 4). Post-hoc comparisons showed that the probability of using at least one visual gesture during the 15-min focal was higher when the subgroup was mainly engaged in social interactions or other behaviours, as compared to feeding/foraging (*p* < 0.001 and *p* = 0.002 for social interactions and other behaviours, respectively), resting (*p* = 0.023 and *p* = 0.035, respectively) or travelling (*p* = 0.001 and *p* = 0.002, respectively; Fig. 4).

**Table 4:** Results of the five models run, with estimates, standard errors (SE), confidence intervals (CIs), likelihood ratio tests (LRT), degrees of freedom (df), and *p* values for each test predictors (marked with an asterisk when significant) and for each control (in italics), with the reference category in parentheses

MODELS	Estimate	SE	2.5% to 97.5% CI	*LRT*	*df*	*p*
**Model 1: Probability of tactile gestures** (GLMM, *χ*^2^ = 6.05, *df* = 6, *p* < 0.001)						
Intercept	−7.92	0.18	−8.28 to −7.56	–	–	–
Signaller’s sex (male)	0.16	0.22	−0.28 to 0.60	0.50	1	0.480
Signaller’s age	−0.31	0.13	−0.57 to −0.04	5.56	1	0.018*
Subgroup activity (other)	0.79	0.51	−0.21 to 1.80	16.86	4	0.002*
Subgroup activity (resting)	−0.28	0.24	−0.74 to 0.19			
Subgroup activity (social interactions)	1.11	0.35	0.43 to 1.78			
Subgroup activity (travelling)	−0.01	0.36	−0.71 to 0.70			
**Model 2: Probability of visual gestures** (GLMM, *χ*^2^ = 40.90, *df* = 6, *p* < 0.001)						
Intercept	−8.19	0.23	−8.63 to −7.75	–	–	–
Signaller’s sex (male)	0.18	0.28	−0.37 to 0.73	0.42	1	0.519
Signaller’s age	−0.09	0.15	−0.38 to 0.20	0.38	1	0.538
Subgroup activity (other)	2.15	0.56	1.04 to 3.25	38.44	4	< 0.001*
Subgroup activity (resting)	0.55	0.24	0.09 to 1.02			
Subgroup activity (social interactions)	1.68	0.37	0.95 to 2.41			
Subgroup activity (travelling)	−0.37	0.43	−1.21 to 0.48			
**Model 4: Probability of accounting for others’ attentional state** (GLMM, *χ*^2^ = 41.78, *df* = 7, *p* < 0.001)						
Intercept	1.71	0.37	0.99 to 2.44	–	–	–
Modality * Signaller’s age	0.47	0.25	−0.02 to 0.96	4.13	1	0.042*
Modality	0.83	0.21	0.42 to 1.25	–	–	–
Signaller’s age	0.01	0.15	−0.28 to 0.31	–	–	–
Vocalization before gesture	2.49	0.72	1.09 to 3.89	7.08	1	< 0.001*
Vocalization after gesture	−0.18	0.65	−1.45 to 1.09	0.08	1	0.777
*Recipient’s age*	−0.27	0.13	−0.51 to −0.02	4.18	1	0.041
*Signaller’s sex (male)*	0.31	0.25	−0.22 to 0.78	1.45	1	0.228
*Recipient’s sex (male)*	−0.29	0.25	−0.79 to 0.20	1.31	1	0.252
*Context (agonistic)*	−0.93	0.59	−2.09 to 0.24	28.74	8	< 0.001
*Context (feeding)*	−1.13	0.48	−2.08 to −0.18			
*Context (fusion)*	1.55	1.09	−0.58 to 3.68			
*Context (resting)*	0.12	0.42	−0.71 to 0.95			
*Context (sexual behaviour)*	−2.19	0.65	−3.46 to −0.91			
*Context (social play)*	0.06	0.36	−0.65 to 0.78			
*Context (solitary play)*	−1.27	0.65	−2.55 to 0.00			
*Context (travelling)*	−0.01	0.88	−1.73 to 1.70			
**Model 5: Probability of being responded to (**GLMM, *χ*^2^ = 15.62, *df* = 3, *p* = 0.001)						
Intercept	4.31	0.69	2.95 to 5.67	–	–	–
Signaller’s age	0.24	0.22	−0.20 to 0.67	1.21	1	0.272
Vocalization before gesture	2.75	1.17	0.45 to 5.04	8.65	1	0.003*
Vocalization after gesture	0.71	1.01	−1.27 to 2.70	0.53	1	0.468
Modality	−0.80	0.32	−1.43 to −0.18	6.58	1	0.010*
*Recipient’s age*	−0.77	0.24	−1.24 to −0.30	10.74	1	0.001
*Signaller’s sex (male)*	0.02	0.45	−0.87 to 0.90	0.00	1	0.969
*Recipient’s sex (male)*	−1.19	0.56	−2.29 to −0.08	4.44	1	0.035
*Context (agonistic)*	−0.68	0.78	−2.21 to 0.84	14.43	8	0.071
*Context (feeding)*	1.54	0.94	−0.31 to 3.39			
*Context (fusion)*	−0.12	0.95	−1.98 to 1.75			
*Context (resting)*	0.01	0.50	−0.98 to 1.00			
*Context (sexual behaviour)*	−1.66	1.01	−3.64 to 0.32			
*Context (social play)*	0.50	0.54	−0.56 to 1.56			
*Context (solitary play)*	−1.84	0.87	−3.56 to −0.13			
*Context (travelling)*	−0.06	1.23	−2.47 to 2.35

*Characteristics of gestural production.* We observed 43 different gesture types, which were produced in many different contexts, including social play (where we observed the use of 33 different gesture types), resting (22), affiliative interactions (19), agonistic interactions (19) and feeding/foraging (18). Please see Table 3 for more details. Gestures were usually performed by a signaller towards a recipient, but they could also involve two individuals interacting with each other while facing a third party, like in the case of arm wrapping (Table 3). On average, individuals produced 10 different gesture types during this study (individual range: 1–24). In Model 3, there was no significant differences between the full and the null models (Table 4), suggesting that repertoire size did not vary depending on signaller’s age. However, this model should be taken with caution, as it was the only one suggesting some problems with the residual distribution. In particular, although QQ plots revealed no significance in the KS distribution test, dispersion test and outlier test, the function plotting residuals against the fitted value showed a humped-shape pattern, suggesting irregularities in the distribution of residuals, which in our case was likely due to the relatively low sample size (*N* = 48).

**Fig. 5:**
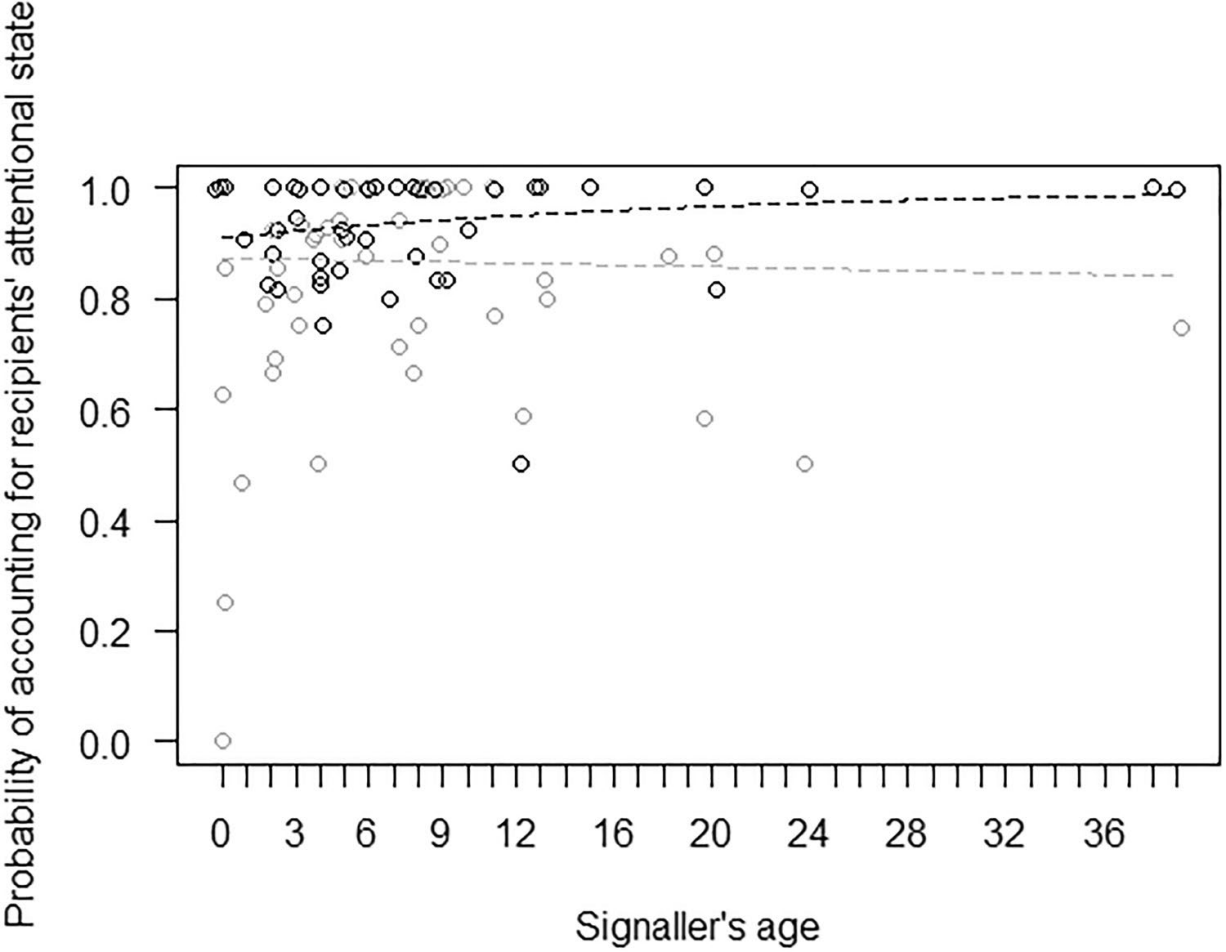
Separately for the tactile modality (in grey) and the visual modality (in black), probability of accounting for recipients’ visual attentional state when producing a gesture, as a function of the signaller’s age (in years; infants: < 2 years; juveniles: 2–5 years; subadults: 6–7 years; adults: > 8 years). Circles represent the mean probability of accounting for recipients’ attentional state for each signaller and modality, after aggregating the data points used for Model 4 (5 individuals were observed in only one modality and the total number of data points is therefore 93). The line represents the fitted model, which was like Model 4, but unconditional on all the factors that were standardized. The probability of accounting for recipients’ attentional state was higher for older individuals, but only for visual gestures, remaining instead similar for tactile gestures

**Fig. 6:**
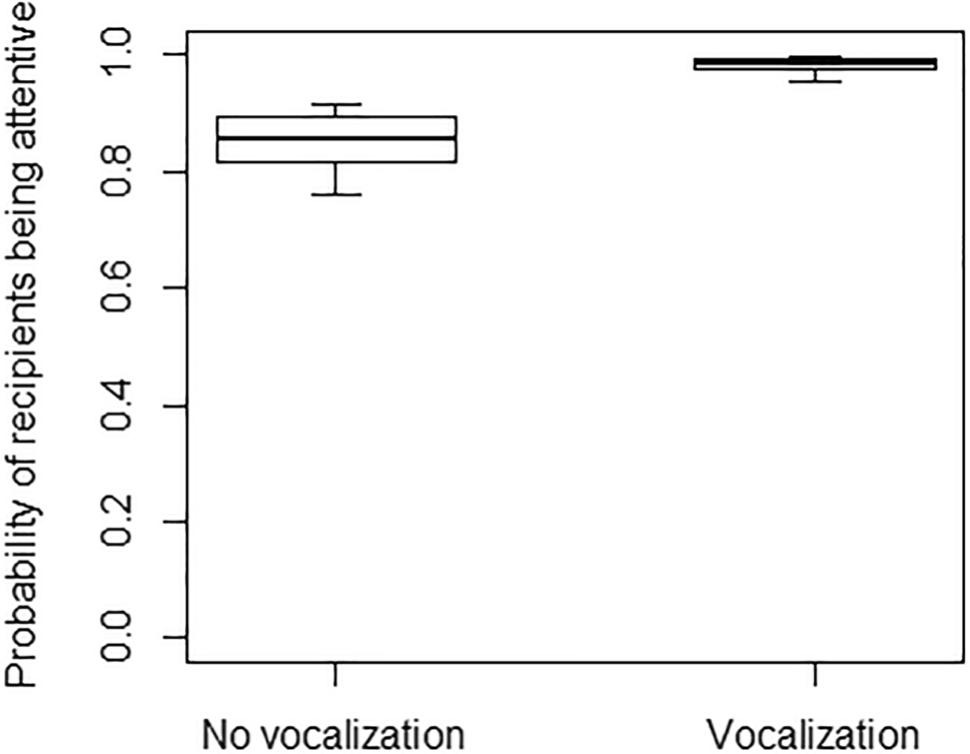
Probability of recipients being attentive, as a function of vocalizations being produced (Vocalization) or not being produced (No vocalizations) in the 2 sec preceding the gesture. The thick lines of the box plots represent the mean probabilities in the two conditions, as estimated by the fitted model, which was like Model 4, but unconditional on all the other factors that were standardized. The ends of the boxes represent the estimated standard errors, and the ends of the whiskers represent the 95% confidence intervals. The probability of recipients being attentive when the gesture was produced was significantly higher when gestures were preceded by a vocalization, as compared to when they were not

**Fig. 7:**
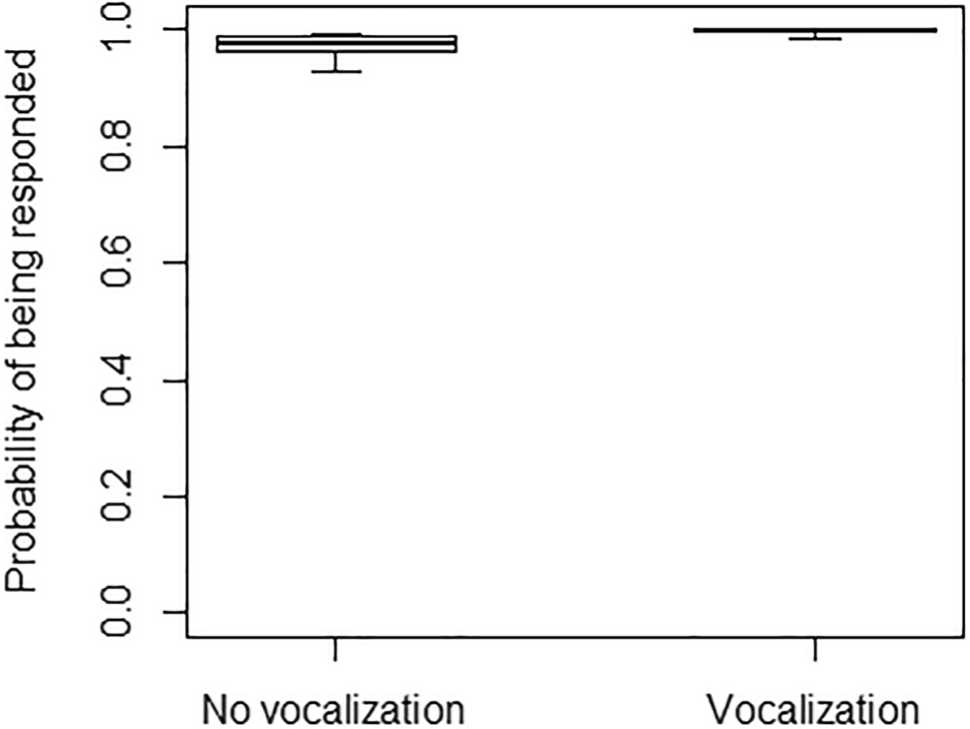
Probability of gestures being responded, as a function of vocalizations being produced (Vocalization) or not being produced (No vocalizations) in the 2 sec preceding the gesture. The thick lines of the box plots represent the mean probabilities in the two conditions, as estimated by the fitted model, which was like Model 4, but unconditional on all the other factors that were standardized. The ends of the boxes represent the estimated standard errors, and the ends of the whiskers represent the 95% confidence intervals. The probability of gestures being responded was significantly higher when gestures were preceded by a vocalization, as compared to when they were not

The probability of accounting for the recipient’s attentional state was relatively high when producing gestures both in the visual modality (91%) and in the tactile one (85%). Tactile gestures were responded 96% of the times (i.e. 648 times), whereas visual gestures were responded 91% of the times (i.e. 468 times). In Model 4, the full model significantly differed from the null model, with the interaction of signaller’s age and gesture modality being significant (Table 4). In particular, the probability of accounting for recipients’ attentional state was higher for older individuals, but only for visual gestures, remaining instead similar for tactile gestures (Fig. 5). Moreover, the probability that the recipient was attentive (when the gesture was produced) was higher when gestures were preceded by a vocalization (Fig. 6), but not when they were followed by a vocalization (Table 4). Finally, in Model 5, the full model significantly differed from the null model, with the probability of being responded increasing when gestures were preceded by a vocalization (Fig. 7), but not when they were followed by a vocalization, and being overall higher for gestures produced in the tactile rather than the visual modality (Table 4).

## Discussion

Our study provides a first description of gestural communication in a Platyrrhine species. The observation of a wild group of spider monkeys revealed the use of a large variety of gestures, which we categorized into 43 different gesture types (Table 3). These gestures were produced in two main modalities (visual and gestural) and in many different contexts. They were usually performed by a signaller towards a recipient, but they could also involve two individuals interacting with each other while facing a third party, like in the case of arm wrapping (Table 3). Our results further showed that younger spider monkeys were more likely than older ones to use tactile gestures within the 15-min focal observations, despite no sex- and age-differences in the probability of producing visual gestures (Table 1). Repertoire size did not vary through age, but the probability of accounting for recipients’ attentional state was higher for older monkeys than for younger ones, especially for visual gestures (Table 1). Using vocalizations right before the gesture increased the probability of gesturing towards attentive recipients and of receiving a response (regardless of the modality in which the gesture was produced), but age had no effect on the probability of gestures being responded (Table 1).

The probability of using tactile gestures within the 15-min focal observations was higher for younger individuals. These results are in line with our predictions (Prediction 1b) and with literature on great apes showing that the proportion of tactile gestures significantly decreases through age in bonobos (*Pan paniscus*), gorillas (*Gorilla gorilla*) and chimpanzees (Schneider et al. 2012). As motility increases through development, immatures increase their distance to their mothers, and tactile gestures between mothers and immatures may become less likely (Schneider et al. 2012). If tactile gestures are mostly directed to mothers, this could explain why their use decreases through age.

However, in contrast to Prediction 2b, we found no developmental changes in the production of visual gestures. These results might be explained in at least two ways. First, Schneider and colleagues (2012) found an increase in the proportion of visual gestures produced before and after 14 months of age. In our study, however, the largest majority of study subjects was older than one year of age, so that we might have failed to detect differences between developmental stages (which, in spider monkeys, might also occur earlier than in apes). Second, Schneider and colleagues (2012) compared the *proportion* of gestures produced through age in the different modalities, so that a decrease in the use of tactile gestures would have automatically also led to an increase in the proportion of visual gestures used. Therefore, it is possible that the use of visual gestures does not increase through development in absolute terms, but only in relation to the use of tactile gestures. Longitudinal approaches (rather than cross-sectional ones) would be surely important to address these open issues. We found no effect of sex on the probability of producing tactile and visual gestures within the 15-min focal observations, in contrast to our Predictions 1a and 2a. However, tactile and visual gestures were used with a different probability depending on the main activity of the subgroup. Both tactile and visual gestures, in particular, were less likely to occur when the subgroup fed/foraged or rested (as compared to when the subgroup engaged in social interactions), but visual gestures were also less likely when the subgroup travelled, and the visual attention of potential recipients might have been lower. As different gesture types might more likely occur in certain contexts, and as in captivity some of these contexts may not be present (e.g. travelling), these results highlight the importance of studying conspecific groups of primates that live in different settings (e.g. wild and captivity) and socio-ecological conditions, to fully understand their communication systems.

In our study, repertoire size did not vary through age. In contrast to Prediction 3, we found no effect of age on the number of different gesture types produced by individuals, neither as linear nor as non-linear relation. This is in contrast with other studies showing developmental changes in repertoire size, with repertoire size either decreasing with age (e.g. Molesti et al. 2020) or peaking in juveniles before declining again (e.g. Call and Tomasello 2007; Hobaiter and Byrne 2011a; Liebal et al. 2004; Schneider et al. 2012). Developmental changes in repertoire size have been extensively studied in other species, because they provide important insights into the emergence of gestural communication. According to the Phylogenetic Ritualization hypothesis, for instance, gestures are largely innate, individual repertoires should be identical at birth across conspecifics, and only contract through age if individuals identify gesture types that are more effective in the specific context they experience, discarding others (e.g. Hobaiter and Byrne 2011a). According to the Ontogenetic Ritualization hypothesis, in contrast, gestures are created by individuals that reciprocally adjust their behaviour during repeated social interactions, so that repertoire size should increase through age (e.g. Call and Tomasello 2007). Therefore, our results are more in line with the Phylogenetic Ritualization hypothesis (and with other studies done in great and small apes, including chimpanzees, bonobos, gorillas and siamangs, *Symphalangus syndactylus*: Amici and Liebal 2022b; Genty et al. 2009; Hobaiter and Byrne 2011a, b), although we found no significant decrease in gestural repertoire size through age.

One reason why we might have failed to detect variation in repertoire size through age is that we did not follow individuals longitudinally, but rather compared the repertoires of individuals having different ages. Given that there might be inter-individual differences in repertoire size, it is possible that developmental changes were masked by these differences. Therefore, caution is needed when interpreting our findings, and more studies with a longitudinal approach are necessary to understand the role played by experience and socialization for the emergence of communication systems (see also Bard et al. 2014; Pika and Fröhlich 2019). Alternatively, it is possible that repertoire size did not change through age because spider monkeys develop their gestural communication system very rapidly and had therefore already reached their adult full repertoire in the very first months of their lives. Longer observational efforts with a developmental perspective will thus be crucial to follow individual developmental patterns of gestural communication, to more reliably monitor whether gestural repertoires change through age also in spider monkeys and when individuals first show the repertoires that will characterize their adult life.

Older monkeys were more likely than younger ones to account for recipients’ attentional states, but only for gestures produced in the visual modality. In line with our Prediction 4a, therefore, spider monkeys, like apes and Cercopithecines (e.g. Amici and Liebal 2022a; Fröhlich et al. 2018; Liebal et al. 2004; Molesti et al. 2020; Schel et al. 2022), differentiate between gestures depending on their modality, and accordingly adjust their production. In our study, the probability of accounting for recipients’ attentional state was relatively high in both modalities (91% for visual gestures, 85% for tactile ones; Fig. 3), in line with literature in species other than great apes, where the largest majority of visual and tactile gestures are produced when recipients are attentive (e.g. 95% of all gestures in red-capped mangabeys: Schel et al. 2022). Crucially, the probability of accounting for recipients’ attentional states, especially in the visual modality, also increased with age, in line with previous findings in great and small apes (e.g. Amici and Liebal 2022a). Through age, individuals may become increasingly exposed to others’ gestures and acquire direct experience about the effectiveness of their own communicative signals. Therefore, they may gradually learn that accounting for others’ visual attention increases the probability of eliciting recipients’ response, without this necessarily implying a cognitive understanding of these processes (see Amici and Liebal 2022a).

Using vocalizations right before the gesture increased the probability that monkeys would gesture towards attentive recipients (in line with our Prediction 4b) and would be responded (in line with our Prediction 5b). When spider monkeys vocalized before gesturing, the probability that recipients would be attentive during gestural production increased from 86% to 98%, whereas the probability of eliciting a response increased from 93% to 99%. Therefore, the multimodal combination of signals might be a powerful tool for spider monkeys to make their communication more effective. These results are partially in line with studies in great apes, which report that individuals may use auditory or tactile attention-getters (e.g. clapping hands or spitting) before visual gesturing, likely to attract recipients’ attention (see Tomasello and Call 2019). In spider monkeys, however, using vocalizations before a gesture increased the effectiveness of their communication regardless of the gesture modality. Therefore, rather than using vocalizations to specifically attract recipients’ attention when necessary (i.e. for visual gestures), spider monkeys might fail to discriminate between the contingencies of the two modalities, and indiscriminately rely on vocalizations to increase the probability that others will react to their gestures. Indeed, vocalizations could even merely reflect signallers’ arousal, which might enhance recipients’ attention towards the signaller and the probability of responding to it. In the future, it would be interesting to further investigate whether the vocalization preceding the gesture serves as an attention-getter, or also has an independent communicative function (e.g. to transmit other information to receivers). In contrast, we found no effect of age on the probability of gestures being responded (in contrast to our Prediction 5a).

Our study had several important limitations. First, we only observed one group of monkeys. However, including more groups would be useful not only to increase the generalizability of our results but also to assess whether gestural repertoires differ across conspecific groups. Inter-group variation in gestural repertoires, for instance, might provide support to the ontogenetic emergence of gestures (e.g. Tomasello and Call 1997, 2019), whereas similarity in gestural repertories across different groups would rather support the hypothesis that gestures are largely innate (e.g. Hobaiter and Byrne 2011a, b). Second, we only observed the study group for six months. Although this time was likely sufficient to make a reliable estimate of the individuals’ repertoires (see Fig. 1), a longer observational effort would be important to also allow the detection of gestures that might happen very rarely. In spider monkeys, for instance, males are known to sporadically conduct raiding parties into the territory of neighbouring groups, during which males silently move on the ground (Aureli et al. 2006). In the future, it would be interesting to monitor whether the frequency of gestures increases during these events, and if males use specific gesture types that we could not detect in other contexts. Third, gestures are likely to trigger specific responses by recipients, and these responses can be used to infer the meaning of gestures (see Hobaiter and Byrne 2014). In this study, we did not systematically address whether specific gesture types are associated to specific responses also in spider monkeys, but future work should surely investigate this topic. Fourth, our study only focused on gestures and body postures that likely served a communicative function, without including vocalizations and facial expressions. However, communication in primates is known to be multicomponent and multimodal, and future studies in this species should ideally include signals produced in different modalities (Liebal et al. 2022; Slocombe et al. 2011). In the same line, it would be interesting to analyse whether some signals are more likely to co-occur than others, and also whether these combinations might acquire novel meanings as compared to the individual signals (see Amici et al. 2022).

Overall, our study provides a first assessment of gestural communication in a Platyrrhine species, and shows that spider monkeys, like apes and Cercopithecines, share with humans several aspects of their communication systems, including large repertoires, variation in the use of tactile and visual gestures, and sensitivity to the attentional state of recipients. Therefore, some properties that were long thought to be necessary prerequisites of human language evolution appear to be widely shared across species, and it is possible that the divide between communication in humans and other animals may be narrower than usually thought.

## Data Availability

Data can be accessed upon reasonable request to the corresponding author.

## References

[CR01] Altmann J (1974) Observational study of behavior: sampling methods. Behaviour 49:227–266 4597405 10.1163/156853974x00534

[CR1] American Society of Primatologists (2001) Principles for the ethical treatment of nonhuman primates. https://www.asp.org/2021/04/20/principles-for-the-ethical-treatment-of-non-human-primates/. Accessed 13 Feb 2023

[CR2] Amici F, Liebal K (2022a) The social dynamics of complex gestural communication in great and lesser apes (*Pan troglodytes, Pongo abelii, Symphalangus syndactylus*). Philos Trans R Soc Lond B Biol Sci 377:20210299 35934967 10.1098/rstb.2021.0299 PMC9358312

[CR3] Amici F, Liebal K (2022b) Testing hypotheses for the emergence of gestural communication in great and small apes (*Pan troglodytes, Pongo abelii, Symphalangus syndactylus*). Int J Primatol. 10.1007/s10764-022-00342-7 10.1098/rstb.2021.0299 PMC9358312 35934967

[CR4] Amici F, Oña L, Liebal K (2022) Compositionality in primate gestural communication and multicomponent signal displays. Int J Primatol. 10.1007/s10764-022-00316-935043025

[CR5] Anderson JR, Kuroshima H, Hattori Y, Fujita K (2010) Flexibility in the use of requesting gestures in squirrel monkeys (*Saimiri sciureus*). Am J Primatol 72:707–714 20568077 10.1002/ajp.20827

[CR6] Arbib MA, Liebal K, Pika S (2008) Primate vocalization, gesture, and the evolution of human language. Curr Anthropol 49:1053–1076 19391445 10.1086/593015

[CR7] Aureli F, Schaffner CM, Verpooten J, Slater K, Ramos-Fernandez G (2006) Raiding parties of male spider monkeys: insights into human warfare? Am J Phys Anthropol 131:486–497 16685723 10.1002/ajpa.20451

[CR8] Aureli F, Schaffner CM, Boesch C, Bearder SK, Call J et al (2008) Fission-fusion dynamics: new research frameworks. Curr Anthropol 49:627–641

[CR9] Aychet J, Pezzino P, Rossard A, Bec P, Blois-Heulin C, Lemasson A (2020) Red-capped mangabeys (*Cercocebus torquatus*) adapt their interspecific gestural communication to the recipient’s behaviour. Sci Rep 10:1–12 32732945 10.1038/s41598-020-69847-6 PMC7393380

[CR10] Baayen RH, Davidson DJ, Bates DM (2008) Mixed-effects modeling with crossed random effects for subjects and items. J Mem Lang 59:390–412

[CR11] Bard KA, Dunbar S, Maguire-Herring V, Veira Y, Hayes KG, McDonald K (2014) Gestures and social-emotional communicative development in chimpanzee infants. Am J Primatol 76:14–29 24038115 10.1002/ajp.22189

[CR12] Beltrán Francés V, Castellano-Navarro A, Illa Maulany R, Oka Ngakan P, MacIntosh AJJ, Llorente M, Amici F (2020) Play behavior in immature moor macaques (*Macaca maura*) and Japanese macaques (*Macaca fuscata*). Am J Primatol 82:23192 10.1002/ajp.23192 32882065

[CR13] Bourjade M, Meguerditchian A, Maille A, Gaunet F, Vauclair J (2014) Olive baboons, *Papio anubis*, adjust their visual and auditory intentional gestures to the visual attention of others. Anim Behav 87:121–128

[CR14] Bradbury JW, Vehrencamp SL (2011) Principles of animal communication. Sinauer Associates, Sunderland

[CR15] Brooks ME, Kristensen K, van Benthem KJ, Magnusson A, Berg CW et al (2017) glmmTMB balances speed and flexibility among packages for zero-inflated generalized linear mixed modeling. R J 9:378–400

[CR16] Bründl AC, Tkaczynski PJ, Nohon Kohou G, Boesch C, Wittig RM, Crockford C (2021) Systematic mapping of developmental milestones in wild chimpanzees. Dev Sci 24:e12988 32412141 10.1111/desc.12988

[CR17] Byrne RW, Cartmill E, Genty E, Graham KE, Hobaiter C, Tanner J (2017) Great ape gestures: intentional communication with a rich set of innate signals. Anim Cogn 20:755–769 28502063 10.1007/s10071-017-1096-4 PMC5486474

[CR18] Call J, Tomasello M (eds) (2007) The gestural communication of apes and monkeys. Lawrence Erlbaum, Mahwah/London

[CR19] Canteloup C, Bovet D, Meunier H (2015) Intentional gestural communication and discrimination of human attentional states in rhesus macaques (*Macaca mulatta*). Anim Cogn 18:875–883 25749401 10.1007/s10071-015-0856-2

[CR20] Cartmill EA, Maestripieri D (2012) Socio-cognitive specializations in non-human primates: evidence from gestural communication. The Oxford handbook of comparative evolutionary psychology. Oxford University Press, Oxford, pp 166–193

[CR21] Castellano-Navarro A, Macanás-Martínez E, Xu Z et al (2021) Japanese Macaques’ (*Macaca fuscata*) sensitivity to human gaze and visual perspective in contexts of threat, cooperation, and competition. Sci Rep 11:5264 33664316 10.1038/s41598-021-84250-5 PMC7933183

[CR22] Defolie C, Malassis R, Serre M, Meunier H (2015) Tufted capuchins (*Cebus apella*) adapt their communicative behaviour to human’s attentional states. Anim Cogn 18:747–755 25630371 10.1007/s10071-015-0841-9

[CR23] Dobson AJ, Barnett AG (2018) An introduction to generalized linear models, 4th edn. Chapman and Hall/CRC, New York

[CR24] Fishbein AR, Fritz JB, Idsardi WJ, Wilkinson GS (2019) What can animal communication teach us about human language? Philos Trans R Soc Lond B Biol Sci 375:20190042 31735148 10.1098/rstb.2019.0042 PMC6895550

[CR25] Freeberg TM, Dunbar RI, Ord TJ (2012) Social complexity as a proximate and ultimate factor in communicative complexity. Philos Trans R Soc B 367:1785–1801 10.1098/rstb.2011.0213 PMC3367695 22641818

[CR26] Fröhlich M, Wittig RM, Pika S (2016) Play-solicitation gestures in chimpanzees in the wild: flexible adjustment to social circumstances and individual matrices. R Soc Open Sci 3(8):160278 27853603 10.1098/rsos.160278 PMC5108953

[CR27] Fröhlich M, Wittig RM, Pika S (2018) The ontogeny of intentional communication in chimpanzees in the wild. Dev Sci 22:e12716 30156360 10.1111/desc.12716

[CR28] Genty E, Breuer T, Hobaiter C, Byrne RW (2009) Gestural communication of the gorilla (*Gorilla gorilla*): repertoire, intentionality and possible origins. Anim Cogn 12:527–546 19184669 10.1007/s10071-009-0213-4 PMC2757608

[CR29] Graham KE, Furuichi T, Byrne RW (2017) The gestural repertoire of the wild bonobo (*Pan paniscus*): a mutually understood communication system. Anim Cogn 20:171–177 27632158 10.1007/s10071-016-1035-9 PMC5306194

[CR30] Gupta S, Sinha A (2016) Not here, there! Possible referential gesturing during allogrooming by wild bonnet macaques, *Macaca radiata*. Anim Cogn 19:1243–1248 27395041 10.1007/s10071-016-1012-3

[CR31] Gupta S, Sinha A (2019) Gestural communication of wild bonnet macaques in the Bandipur National Park. Southern India Behav Proc 168:103956 10.1016/j.beproc.2019.103956 31493494

[CR32] Hartig F (2022) DHARMa: residual diagnostics for hierarchical (multi-level/mixed) regression models. R package version 0.4.5. Available online: https://cran.r-project.org/package=DHARMa

[CR33] Hesler N, Fischer J (2007) Gestural communication in Barbary macaques (*Macaca sylvanus*): an overview. In: Call J, Tomasello M (eds) The gestural communication of apes and monkeys. Lawrence Erlbaum, Mahwah/London

[CR34] Hobaiter C, Byrne R (2011a) The gestural repertoire of the wild chimpanzee. Anim Cogn 14:745–767 21533821 10.1007/s10071-011-0409-2

[CR35] Hobaiter C, Byrne R (2011b) Serial gesturing by wild chimpanzees: its nature and function for communication. Anim Cogn 14:827–838 21562816 10.1007/s10071-011-0416-3

[CR36] Hobaiter C, Byrne RW (2014) The meanings of chimpanzee gestures. Curr Biol 24:1596–1600 24998524 10.1016/j.cub.2014.05.066

[CR37] Hobaiter K, Byrne RW (2017) What is a gesture? A meaning-based approach to defining gestural repertoires. Neurosci Biobehav Rev 82:3–12 29229064 10.1016/j.neubiorev.2017.03.008

[CR38] Hockett CF (1960) Logical considerations in the study of animal communication. In: Lanyon WE, Tavolga WN (eds) Animal sounds and communication. American Institute of Biological Sciences, Washington, DC, pp 392–430

[CR39] Lenth R (2020) emmeans: estimated marginal means, aka least-squares means. R package version 1.5.0. See https://CRAN.R-project.org/package=emmeans

[CR40] Liebal K, Call J (2012) The origins of non-human primates’ manual gestures. Philos Trans R Soc Lond B Biol Sci 367:118–128 22106431 10.1098/rstb.2011.0044 PMC3223783

[CR41] Liebal K, Pika S, Tomasello M (2004) Social communication in siamangs (*Symphalangus syndactylus*): use of gestures and facial expressions. Primates 45:41–57 14655035 10.1007/s10329-003-0063-7

[CR42] Liebal K, Pika S, Tomasello M (2006) Gestural communication of orangutans (*Pongo pygmaeus*). Gesture 6:1–38

[CR43] Liebal K, Müller C, Pika S (2007) Gestural communication in nonhuman and human primates. John Benjamins Publishing Company, Amsterdam

[CR44] Liebal K, Waller B, Burrows A, Slocombe K (2013) Primate communication: a multimodal approach. Cambridge University Press, Cambridge 10.1177/147470491301100305 PMC10480985 23864293

[CR45] Liebal K, Slocombe KE, Waller BM (2022) The language void 10 years on: multimodal primate communication research is still uncommon. Ethol Ecol Evol 34:1–14

[CR46] Lüdecke D, Ben-Shachar M, Patil I, Waggoner P, Makowski D (2021) Performance: an R Package for assessment, comparison and testing of statistical models. J Open Source Softw 6:3139

[CR47] Maestripieri D (1996a) Gestural communication and its cognitive implications in pigtail macaques (*Macaca nemestrina*). Behaviour 133:997–1022

[CR48] Maestripieri D (1996b) Social communication among captive stump-tailed macaques (*Macaca arctoides*). Int J Primatol 17:785–802

[CR49] Maestripieri D (1997) Gestural communication in macaques: usage and meaning of nonvocal signals. Evol Comm 1:193–222

[CR50] Meunier H, Fizet J, Vauclair J (2013) Tonkean macaques communicate with their right hand. Brain Lang 126:181–187 23748098 10.1016/j.bandl.2013.05.004

[CR51] Miles J (2005) Tolerance and variance inflation factor. In: Encyclopedia of statistics in behavioral science. 10.1002/0470013192.bsa683

[CR52] Molesti S, Meguerditchian A, Bourjade M (2020) Gestural communication in olive baboons (*Papio anubis*): repertoire and intentionality. Anim Cogn 23:19–40 31605248 10.1007/s10071-019-01312-y

[CR53] Pika S (2008) What is the nature of the gestural communication of great apes. In: Zlatev J, Racine T, Sinha C, Itkonen E (eds) The shared mind. Perspectives on intersubjectivity. John Benjamins Publishing Company, Amsterdam, pp 165–186

[CR54] Pika S, Fröhlich M (2019) Gestural acquisition in great apes: the social negotiation hypothesis. Anim Cogn 22:551–565 29368287 10.1007/s10071-017-1159-6 PMC6647412

[CR55] Pika S, Liebal K (2012) Developments in primate gesture research. John Benjamins Publishing, Amsterdam

[CR56] Prieur J, Barbu S, Blois-Heulin C, Lemasson A (2020) The origins of gestures and language: history, current advances and proposed theories. Biol Rev 95:531–554 31854102 10.1111/brv.12576

[CR57] R Core Team (2020) R: a language and environment for statistical computing. R Foundation for Statistical Computing, Vienna

[CR58] Ramos-Fernández G, Ayala-Orozco B (2003) Population size and habitat use of spider monkeys at Punta Laguna, Mexico. In: Marsh LK (ed) Primates in fragments: ecology and conservation. Kluwer Academic, New York, pp 191–210

[CR59] Roberts SGB, Roberts AI (2019) Social and ecological complexity is associated with gestural repertoire size of wild chimpanzees. Integr Zool 15:276–292 10.1111/1749-4877.12423 PMC7383666 31773892

[CR60] Roberts AI, Roberts SG, Vick SJ (2014) The repertoire and intentionality of gestural communication in wild chimpanzees. Anim Cogn 17:317–336 23999801 10.1007/s10071-013-0664-5

[CR61] Schaffner CM, Slater KY, Aureli F (2012) Age related variation in male–male relationships in wild spider monkeys (*Ateles geoffroyi yucatanensis*). Primates 53:49–56 21881958 10.1007/s10329-011-0271-5

[CR62] Schel AM, Bono A, Aychet J, Pika S, Lemasson A (2022) Intentional gestural communication amongst red-capped mangabeys (*Cercocebus torquatus*). Anim Cogn 25:1313–1330 35362785 10.1007/s10071-022-01615-7 PMC9617956

[CR63] Schneider C, Call J, Liebal K (2012) Onset and early use of gestural communication in nonhuman great apes. Am J Primatol 74:102–113 22025273 10.1002/ajp.21011

[CR64] Shimooka Y, Campbell CJ, Di Fiore A, Felton AM, Izawa K et al (2008) Demography and group composition of Ateles. In: Campbell CJ (ed) Spider monkeys: behavior, ecology and evolution of the genus Ateles. Cambridge University Press, Cambridge, pp 329–348

[CR65] Slocombe KE, Waller BM, Liebal K (2011) The language void: the need for multimodality in primate communication research. Anim Behav 81:919–924

[CR66] Soben C, Llorente M, Villariezo P, Liebal K, Amici F (2023) Maternal investment fosters male but not female social interactions with other group members in immature wild spider monkeys (*Ateles geoffroyi*). Animals 13:1802 37889718 10.3390/ani13111802 PMC10251948

[CR67] Tomasello M, Call J (1997) Primate cognition. Oxford University Press, New York

[CR02] Tomasello M, Call J (2007) Intentional communication in nonhuman primates. In: Call J, Tomasello M (eds) The gestural communication of apes and monkeys. Lawrence Erlbaum Associates, Mahwah, pp 1–15

[CR68] Tomasello M, Call J (2019) Thirty years of great ape gestures. Anim Cogn 22:461–469 29468285 10.1007/s10071-018-1167-1 PMC6647417

[CR69] Tomasello M, George BL, Kruger AC, Farrar MJ, Evans A (1985) The development of gestural communication in young chimpanzees. J Hum Evol 14:175–186

[CR70] Tomasello M, Call J, Nagell K, Olguin R, Carpenter M (1994) The learning and the use of gestural signals by young chimpanzees: a trans-generational study. Primates 35:137–154

